# Hypermethylation of Ring finger protein 41 promoter is associated with early hepatitis B virus-related cirrhosis

**DOI:** 10.3389/fmed.2025.1631990

**Published:** 2025-08-08

**Authors:** Hanxu Zhu, Feng Zhang, Zhezhe Tian, Miaomiao Xu, Yuchen Fan, Shuai Gao, Kai Wang

**Affiliations:** ^1^Department of Hepatology, Qilu Hospital of Shandong University, Jinan, Shandong, China; ^2^Hepatology Institute of Shandong University, Jinan, Shandong, China

**Keywords:** Ring finger protein 41, hepatitis B, liver cirrhosis, DNA methylation, early diagnosis

## Abstract

**Objectives:**

New biomarkers are needed to detect liver cirrhosis at an earlier stage and to individualize treatment strategies. This study specifically investigates the diagnostic potential of Ring finger protein 41 promoter methylation as an epigenetic biomarker for detecting early-stage hepatitis B virus-related liver cirrhosis.

**Methods:**

The methylation level of the Ring finger protein 41 promoter in peripheral blood mononuclear cells of 190 participants were quantified with Methylight, and the changes of serum inflammatory cytokines related to liver fibrosis were analyzed simultaneously.

**Results:**

Patients with early-stage liver cirrhosis exhibited significantly higher methylation levels of Ring finger protein 41 promoter than chronic hepatitis B patients and health controls, accompanied by reduced mRNA expression. Remarkably, the receiver operating characteristic analysis demonstrated that Ring finger protein 41 promoter methylation achieved a superior diagnostic performance (area under the curve) for distinguishing hepatitis B virus-related compensated liver cirrhosis, outperforming conventional non-invasive indicators including liver stiffness measurement, aspartate aminotransferase-to-platelet ratio index, and fibrosis-4 score.

**Conclusion:**

Ring finger protein 41 may play a critical role in the pathogenesis of liver cirrhosis, with its methylation status in peripheral blood mononuclear cells demonstrating strong potential as a non-invasive biomarker for early liver cirrhosis detection.

## 1 Introduction

Liver cirrhosis (LC) is a common chronic progressive liver disease in clinical practice, triggered by one or multiple causative factors. Pathologically, LC shows excessive collagen deposition and other extracellular matrix proteins, scarring, and replacement of normal liver structure by regenerative nodules, potentially leading to organ failure in cirrhosis (1, 2). Despite the increased coverage of hepatitis B vaccination and the availability of effective antiviral drugs, chronic hepatitis B virus (HBV) infection remains the most significant risk factor for the occurrence of LC in Asia (3). Decompensated cirrhosis is characterized by portal hypertension and hepatic insufficiency, and can lead to severe complications, among which gastrointestinal bleeding, ascites, hepatic encephalopathy, and carcinogenesis are the most common. In contrast, compensated cirrhosis typically has no specific clinical symptoms or signs, and liver function test results are often unremarkable, making diagnosis challenging. Recent research evidence indicates that compensated cirrhosis caused by chronic HBV infection can be reversed by suppressing HBV (4, 5). Thus, timely diagnosis of early-stage LC and immediate initiation of antifibrotic therapy can significantly improve the prognosis of patients.

Currently, an accurate diagnosis of LC requires a liver biopsy (6). However, this invasive procedure can cause severe complications in 1% of cases, such as bleeding, severe pain, biliary tract injury, etc. (7, 8). Therefore, a variety of non-invasive methods have been adopted in clinical practice for diagnosis, including transient elastography (TE) and serum biomarker detection. Liver stiffness measurement (LSM) provided by TE is highly regarded in clinical settings due to its distinct advantages of being non-invasive, highly repeatable, and highly objective (9, 10). Nevertheless, its accuracy can be influenced by a multitude of factors, such as obesity, the presence of ascites, and the proficiency of the operating technique (11). Although widely used in clinical practice, the fibrosis-4 score (FIB-4) and aspartate aminotransferase-to-platelet ratio index (APRI) is limited by a high rate of false positives and false negatives (12, 13). As a result, there is an urgent need for a more effective biomarker to improve the accuracy and timeliness of the early diagnosis of LC. Circulating epigenetic markers like CXCR4 methylation (14) and specific CpG sites (15) show promise for non-invasive liver fibrosis detection, though most require further validation in biopsy-proven cohorts.

Ring finger protein 41 (RNF41), namely neuregulin receptor degradation protein 1 (Nrdp1), is a crucial E3 ubiquitin-protein ligase that can specifically degrade a variety of proinflammatory cytokine receptors, adaptors, and kinases through ubiquitination (16, 17). Moreno-Lanceta et al. revealed a pivotal role of RNF41 in hepatic fibrosis and regeneration. Compared with HC, RNF41 expression in macrophages was significantly downregulated in both cirrhotic patients and mice models of hepatic fibrosis. Through the development of a nanoparticle-based delivery system, they demonstrated that RNF41 ameliorates liver fibrosis and reduces hepatic injury by suppressing hepatic stellate cell (HSC) activation signaling while upregulating matrix metalloproteinase-9 (MMP-9) and insulin-like growth factor-1 (IGF-1) (18). Additionally, RNF41 was found to promote macrophage polarization toward an anti-inflammatory phenotype. The binding and ubiquitination of MyD88 by RNF41 can indirectly attenuate the activation of NF-κB. Consequently, this leads to a reduction in the transcription and release of pro-inflammatory cytokines such as tumor necrosis factor-α (TNF-α) and interleukin-6 (IL-6), thereby alleviating the inflammatory response (19–21).

In recent years, epigenetic mechanisms have garnered substantial scientific attention as a pivotal regulatory paradigm that governs gene expression without altering DNA sequences (22). Among these mechanisms, DNA methylation has emerged as a cornerstone of epigenetic regulation, functioning through the covalent addition of a methyl group to the 5′-carbon position of cytosine residues within CpG dinucleotides (23–25). Accumulating evidence demonstrates that DNA methylation of key regulatory genes, including SUN2, PTCH1, PSTPIP2, and PTEN, plays a crucial role in hepatic stellate cell (HSC) activation and subsequent promotion of liver fibrosis progression (26). Therefore, methylation is expected to provide a new way to diagnose liver fibrosis, thereby improving patient prognosis.

RNF41 promoter methylation status as a biomarker has not, to our knowledge, been evaluated. Therefore, the study aims to (1) investigate the mRNA expression profile and promoter methylation status of RNF41 in peripheral blood mononuclear cells (PBMCs) from HBV-related cirrhotic patients, (2) elucidate the clinical-pathological correlations between RNF41 promoter hypermethylation and disease progression markers, and (3) systematically evaluate the diagnostic superiority of RNF41 methylation levels over conventional noninvasive indices—including LSM, APRI, FIB-4 score—for cirrhosis staging and therapeutic monitoring. These investigations seek to establish an epigenetics-based paradigm for early cirrhosis detection while providing mechanistic insights for personalized therapeutic strategies.

## 2 Methods

### 2.1 Patients

**Figure 1:**
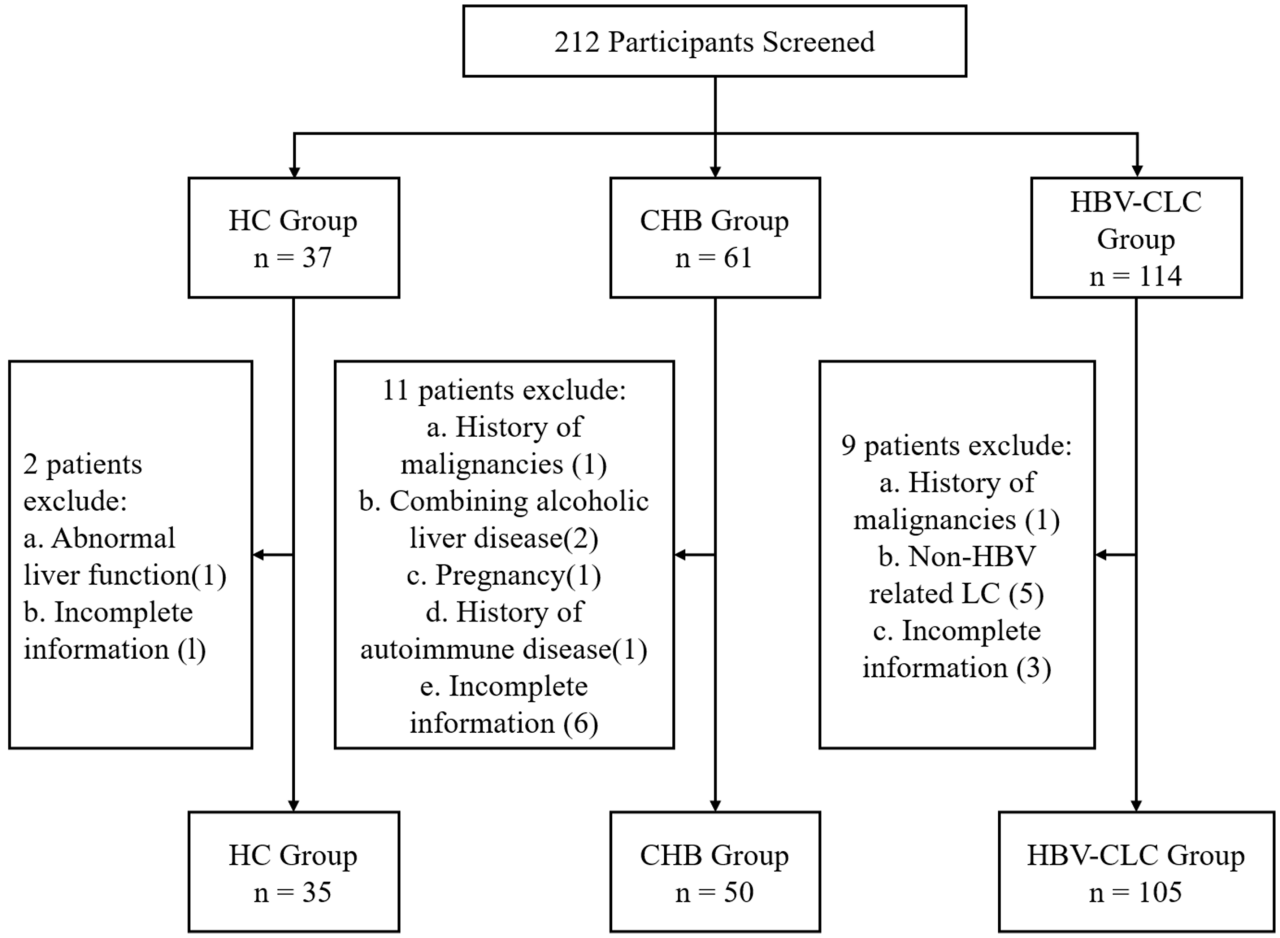
Study enrollment flowchart. Boxes indicate the number of participants at each stage, with exclusion criteria and corresponding numbers shown for screened but excluded individuals.

From November 2023 to January 2025, we retrospectively enrolled 190 patients from the Department of Hepatology, Qilu Hospital of Shandong University, including 35 health controls (HC), 50 chronic hepatitis B (CHB) patients, and 105 HBV-CLC cases. CHB patients were enrolled according to the 2018 AASLD Practice Guidelines, meeting the following criteria: 1. Hepatitis B surface antigen (HBsAg) positivity for ≥6 months at enrollment; 2. No evidence of cirrhosis based on clinical, biochemical, and imaging evaluations. All HBV-CLC patients were enrolled according to the 2020 Chinese Guidelines for Cirrhosis Management, meeting the following diagnostic criteria: 1. HBsAg positivity for ≥6 months; 2. Cirrhosis confirmed by histopathology, endoscopy, or imaging (ultrasound, CT or MRI); 3. At least two of the following laboratory abnormalities: platelet count <100 × 10^9^/L, serum albumin <35 g/L, international normalized ratio (INR) > 1.3, prolonged prothrombin time, or APRI score >2. 4. no obvious signs of decompensated liver cirrhosis. The patient selection process and exclusion criteria are detailed in Figure 1. All participants were recruited following strict inclusion/exclusion criteria and provided written informed consent. The study protocol was approved by the Medical Ethics Committee of Qilu Hospital, Shandong University.

### 2.2 Sodium bisulfite of DNA modification

Peripheral blood samples were collected in citrate anticoagulant tubes from all participants. PBMCs were isolated using Ficoll-Paque Plus (GE Healthcare, Uppsala, Sweden) density gradient centrifugation. Genomic DNA was extracted from PBMCs using TRIzol Reagent (Invitrogen, Carlsbad, CA, USA), followed by bisulfite conversion with the EZ DNA Methylation-Gold Kit (Zymo Research, Orange, CA, USA) to ensure complete cytosine conversion.

### 2.3 TaqMan probe-based quantitative methylation-specific PCR

Specific primers and probes were designed for both the bisulfite-converted DNA templates of target gene RNF41 and reference gene ACTB. The complete sequences, including forward and reverse primers along with dual-labeled TaqMan probes, are detailed in Table 1. Thermal cycling was performed following the manufacturer’s standard protocol: initial denaturation at 95°C for 15 min, followed by 45 cycles of 95°C for 15 s and 60°C for 1 min. Methylation levels were quantified using the percentage of methylated reference value (PMR), calculated as follows:


$$\mathrm{PMR}=100\%\times 2^{-[\begin{array}{l} \mathrm{\Delta CT}(\text{target gene}-\text{control gene})\text{Sample}-\mathrm{\Delta CT}\\ (\text{target gene}-\text{control gene})M.\text{SssI}-\text{Reference} \end{array}]}$$

**Table 1:** Sequences of used primers and probes.

Gene	Forward primer sequence (5′-3′)	Reverse primer sequence (5′-3′)	Probe oligo sequence
Methylight			
RNF41	GGTTGGTTTCGGATTTATGATTTTA	AACAAAACTCCTTTACCGAAATAA	ACCAAATACAACGACTCACGCCTATAATCCT
ACTB	TGGTGATGGAGGAGGTTTAGTAAGT	AACCAATAAAACCTACTCCTCCCTTAAA	ACCACCACCCAACACACAATAACAAACACA
RT-qPCR			
RNF41	ATCGCAGAGCTGGAGAAGACGT	TGTCTCCTCCAGGTTCTGAAGG	
β-actin	CATGTACGTTGCTATCCAGGC	CTCCTTAATGTCACGCACGAT	

### 2.4 RNA extraction and quantitative real-time polymerase chain reaction

Total RNA was extracted from PBMCs using TRIzol Reagent and immediately reverse transcribed into cDNA using the First Strand cDNA Synthesis Kit (Fermentas, Vilnius, Lithuania). RT-qPCR was performed in a 10 μL reaction volume according to the manufacturer’s protocol, with the following cycling conditions: initial denaturation at 95°C for 30 s, followed by 45 cycles of 95°C for 5 s, 60°C for 30 s, and 72°C for 30 s. The fold changes were quantified by the 2^−∆∆Ct^ method. The specific primer sequences for RNF41 and the reference gene ACTB are provided in Table 1.

### 2.5 Enzyme-linked immunosorbent assay (ELISA)

Plasma levels of TNF-α, Interleukin-10 (IL-10), and transforming growth factor-β (TGF-β) were quantified using the Human Immunoassay Valukine ELISA Kit (Lengton Bioscience Co., Shanghai, China) according to the manufacturer’s protocol. Absorbance was measured at 450 nm using a microplate reader, with all samples analyzed in duplicate to ensure data reproducibility.

### 2.6 Clinical data collection

Comprehensive clinical and laboratory data were collected for all participants, including age, sex, HBV virological markers, liver function, coagulation profile, complete blood count, LSM, and abdominal imaging findings. Liver fibrosis indices were calculated using standard formulas:


$$\mathrm{FIB}-4=(\mathrm{Age}\times \mathrm{AST})/(\text{Platelet Count}(10^{9}∕L)\times \sqrt{\mathrm{ALT}})$$

$$\begin{array}{l} \text{APRI}=[(\mathrm{AST}\;\text{level}/\text{upper limit of normal})\times 100]\\ ∕\text{Platelet Count}(10^{9}∕L) \end{array}$$

### 2.7 Statistical analysis

Statistical analyses were performed using SPSS version 26.0 statistical software (SPSS Inc., Chicago, IL, USA). Quantitative variables are expressed as median (centile 25; centile 75). Categorical variables were expressed as numbers (%). Categorical variables were compared using Pearson’s chi-square test. Mann–Whitney U test and Kruskal-Wallis H test were used to compare the quantitative variables. The Spearman rank correlation test was used to analyze the relationship between RNF41 methylation and quantitative clinical data as well as TL1A mRNA expression level, and serum cytokines expression. The receiver operating characteristic (ROC) curve was performed to assess the diagnostic value of RNF41 methylation level and a model based on binary logistic regression was established to estimate the diagnostic value of RNF41 methylation level. The optimal cut-off point was defined as the point that maximizes sensitivity plus specificity. *p* < 0.05 was considered to be statistically significant.

## 3 Results

### 3.1 General characteristics of the study populations

The study cohort comprised 190 participants stratified into three diagnostic categories: 105 patients with HBV-CLC, 50 CHB patients, and 35 HCs. Comprehensive baseline characteristics, including demographic profiles, clinical manifestations, and serological biomarkers, were systematically analyzed and tabulated in Table 2.

**Table 2:** General clinical characteristics of the patients.

Variables	HC (*n* = 35)	CHB (*n* = 50)	HBV-CLC (*n* = 105)	*p* Value
Age	52.00 (37.00–60.00)	50.00 (37.75–58.25)	52.00 (45.00–57.00)	0.390^b^
Gender, Male (%)	22.00 (62.86)	32.00 (64.00)	72.00 (68.57)	0.761^a^
Log10[HBV DNA]	NA	3.26 (2.77–4.71)	2.88 (2.12–4.75)	0.259^c^
HBsAg (IU/L)	NA	2,671.31 (874.59–5,855.88)	1,015.56 (450.32–2,353.37)	0.002^c^
HBeAg, + (%)	NA	23.00 (46.00)	41.00 (39.05)	0.486^a^
ALT (IU/L)	17.00 (14.00–28.00)	19.00 (15.00–28.25)	25.00 (20.00–33.50)	0.001^b^
AST (IU/L)	19.00 (14.00–23.00)	22.00 (17.00–26.25)	28.00 (22.00–36.00)	<0.001^b^
TBIL (μmol/L)	10.40 (7.60–13.20)	10.50 (7.60–13.58)	16.10 (11.60–22.00)	<0.001^b^
ALB (g/L)	46.00 (42.50–47.60)	47.10 (45.80–49.10)	46.00 (43.50–47.70)	0.003^b^
PTA (%)	113.00 (94.00–119.00)	101.00 (90.75–106.50)	90.00 (78.00–99.00)	<0.001^b^
INR	0.93 (0.89–1.02)	0.99 (0.94–1.05)	1.05 (1.00–1.14)	<0.001^b^
PLT (×10^12/L)	236.00 (200.00–273.00)	190.50 (150.75–219.25)	124.00 (83.00–167.50)	<0.001^b^
AFP (ng/mL)	NA	2.57 (1.67–3.20)	2.79 (1.97–5.03)	0.04^c^
LSM (kPa)	NA	6.50 (5.00–8.15)	12.00 (8.95–17.30)	<0.001^c^
FIB-4	0.81 (0.58–1.38)	1.21 (0.78–1.83)	2.40 (1.28–4.02)	<0.001^b^
APRI	0.19 (0.16–0.26)	0.27 (0.21–0.38)	0.62 (0.35–1.10)	<0.001^b^

^a^Pearson’s chi-square test; ^b^Kruska-Wallis H test; ^c^Mann–Whitney U test.

### 3.2 Methylation status and mRNA expression of RNF41 in LC, CHB, and HCs

**Figure 2:**
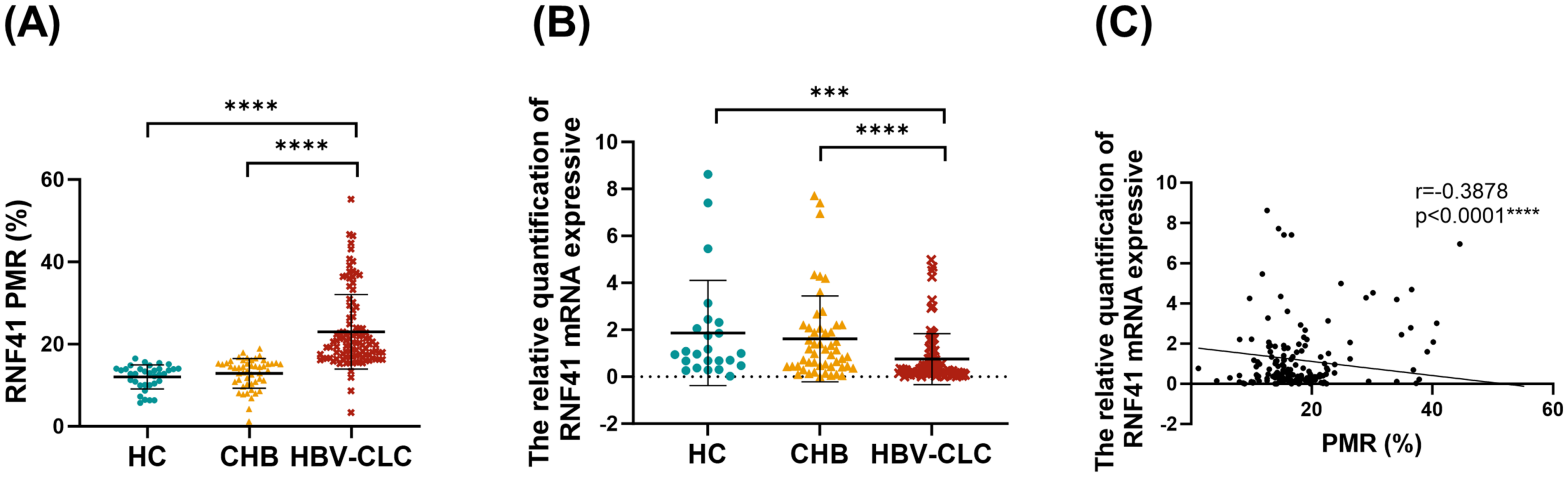
Comparison of RNF41 PMR values and mRNA expression in health controls, chronic hepatitis B and hepatitis B virus-related compensated cirrhosis. **(A)** Methylight showing RNF41 methylation levels in PBMCs from health controls and patients with CHB and hepatitis B virus-related compensated cirrhosis. **(B)** Levels of RNF41 mRNA in HCs and patients with CHB and HBV-related compensated cirrhosis were measured with quantitative real-time polymerase chain reaction. **(C)** Association between the methylation and mRNA levels of the RNF41 promoter in PBMCs (Spearman’s *r* = −0.3878, *p* < 0.001). Statistical analysis of PMR **(A)** and mRNA expression **(B)** based on the mean ± SD of at least three independent experiments. *p* values were obtained using Student’s t test. *p* < 0.005 is indicated by ***, and *p* < 0.001 is indicated by ****.

The MethyLight method was employed to perform a quantitative analysis of RNF41 promoter methylation status in PBMCs across HC, CHB, and HBV-CLC. PMR values were, respectively, determined and graphically represented in Figure 2A. RNF41 methylation levels are significantly higher in patients with HBV-CLC (median 19.98%, interquartile range 16.85–25.6%) than in those with CHB (median 14.23%, interquartile range 10.67–15.31%, *p* < 0.0001) and HC (median 12.77%, interquartile range 10.06–13.96%, *p* < 0.0001). However, there were no significant differences between the CHB and HC groups (*p* = 0.0793). Figure 2B illustrates a significant downregulation of RNF41 mRNA expression was observed in HBV-CLC (median 0.302, interquartile range 0.136–0.783) compared to CHB (median 1.143, interquartile range 0.448–2.163) and HC (median 0.981, interquartile range 0.675–2.018), demonstrating transcriptional suppression during cirrhosis progression. It was found that CHB and HC groups were not significantly different. Additionally, spearman rank correlation analysis between the PMR values and mRNA expression of RNF41 was performed, revealing a negative correlation (Figure 2C).

### 3.3 Association between PMR values of RNF41 and clinical features

**Figure 3:**
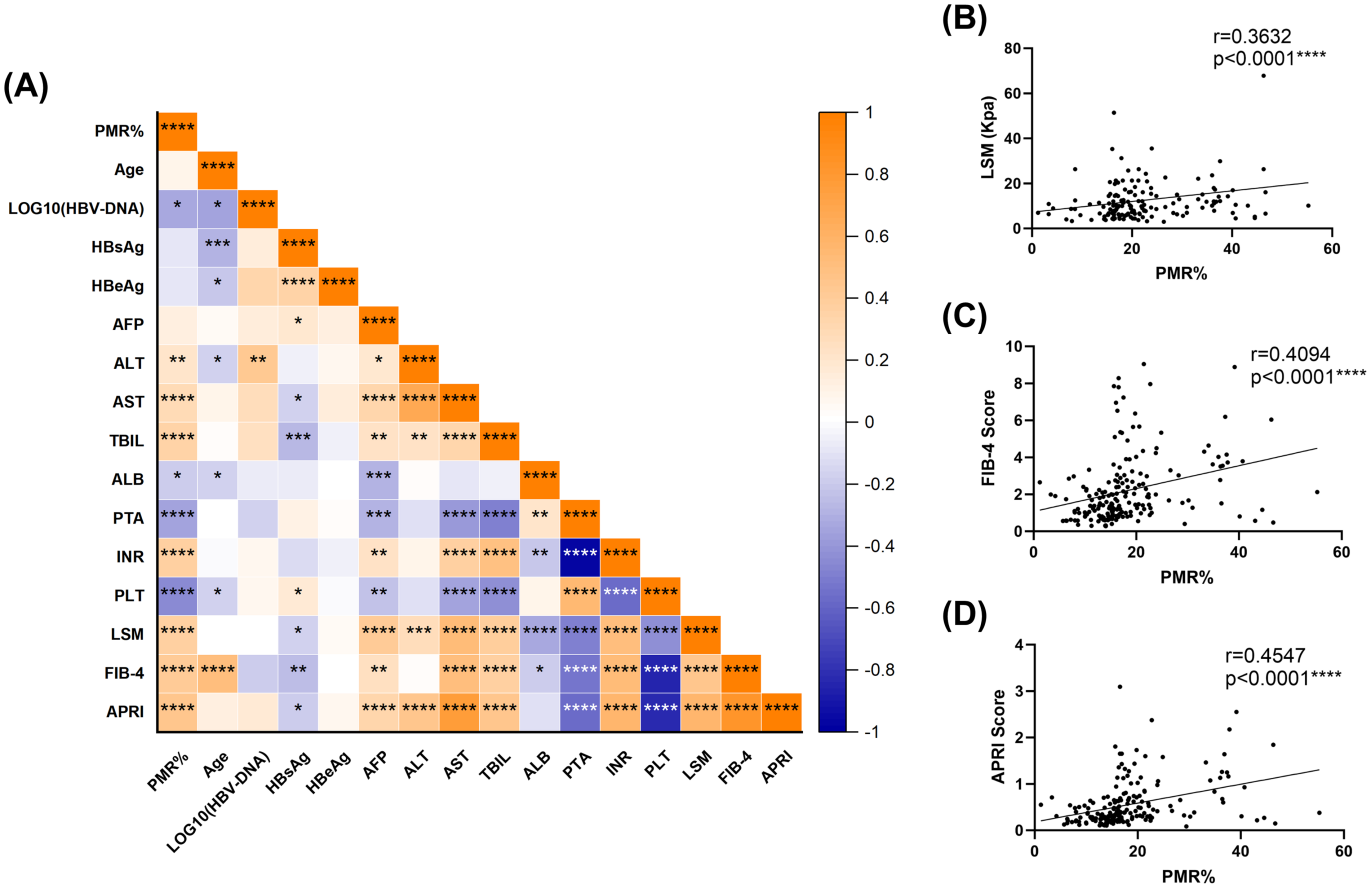
Relationships between RNF41 methylation level and clinical features. **(A)** Heatmap of correlation between the clinical indicators of patients. **(B–D)** Spearman correlation analysis revealed a significant positive correlation between RNF41 PMR and conventional non-invasive fibrosis markers, including LSM, FIB-4, and APRI. (Spearman’s *r* = 0.3632, 0.4094, 0.4547, *p* < 0.0001, respectively). *p < 0.05, **p ≤ 0.01, ***p ≤ 0.001, ****p ≤ 0.0001.

To evaluate the diagnostic potential of RNF41 methylation as a novel biomarker for HBV-related early cirrhosis, comprehensive Spearman correlation analyses were performed between RNF41 PMR levels and clinical parameters, including liver fibrosis indices, across CHB and HBV-CLC cohorts. As illustrated in the correlation heatmap (Figure 3A), RNF41 PMR exhibited significant positive correlations with alanine aminotransferase (ALT) (*r* = 0.2129, *p* = 0.0024), aspartate aminotransferase (AST) (*r* = 0.2928, *p* < 0.0001), total bilirubin (TBIL) (*r* = 0.3435, *p* < 0.0001) and INR (*r* = 0.3646, *p* < 0.0001). Conversely, inverse correlations were observed with viral load [log10(HBV-DNA); *r* = −0.3292, *p* = 0.0499], albumin (ALB) (*r* = −0.1861, *p* < 0.0101), prothrombin time activity (PTA) (*r* = −0.3490, *p* < 0.0001), and platelet (PLT) (*r* = −0.4550, *p* < 0.0001). Notably, no significant associations were detected with age, HBsAg titer, HBeAg status, or alpha-fetoprotein (AFP). Furthermore, we systematically evaluated the clinical relevance of RNF41 methylation status by analyzing its correlation with established non-invasive fibrosis indices (Figures 3B–D). Significant associations were identified between RNF41 PMR values and LSM (*r* = −0.3632, *p* < 0.0001), FIB-4 score (*r* = −0.4094, *p* < 0.0001), and APRI (*r* = −0.4547, *p* < 0.0001). These correlations suggest the potential utility of RNF41 methylation as a complementary biomarker for early liver fibrosis.

### 3.4 Related cytokine expression levels change in different groups

**Figure 4:**
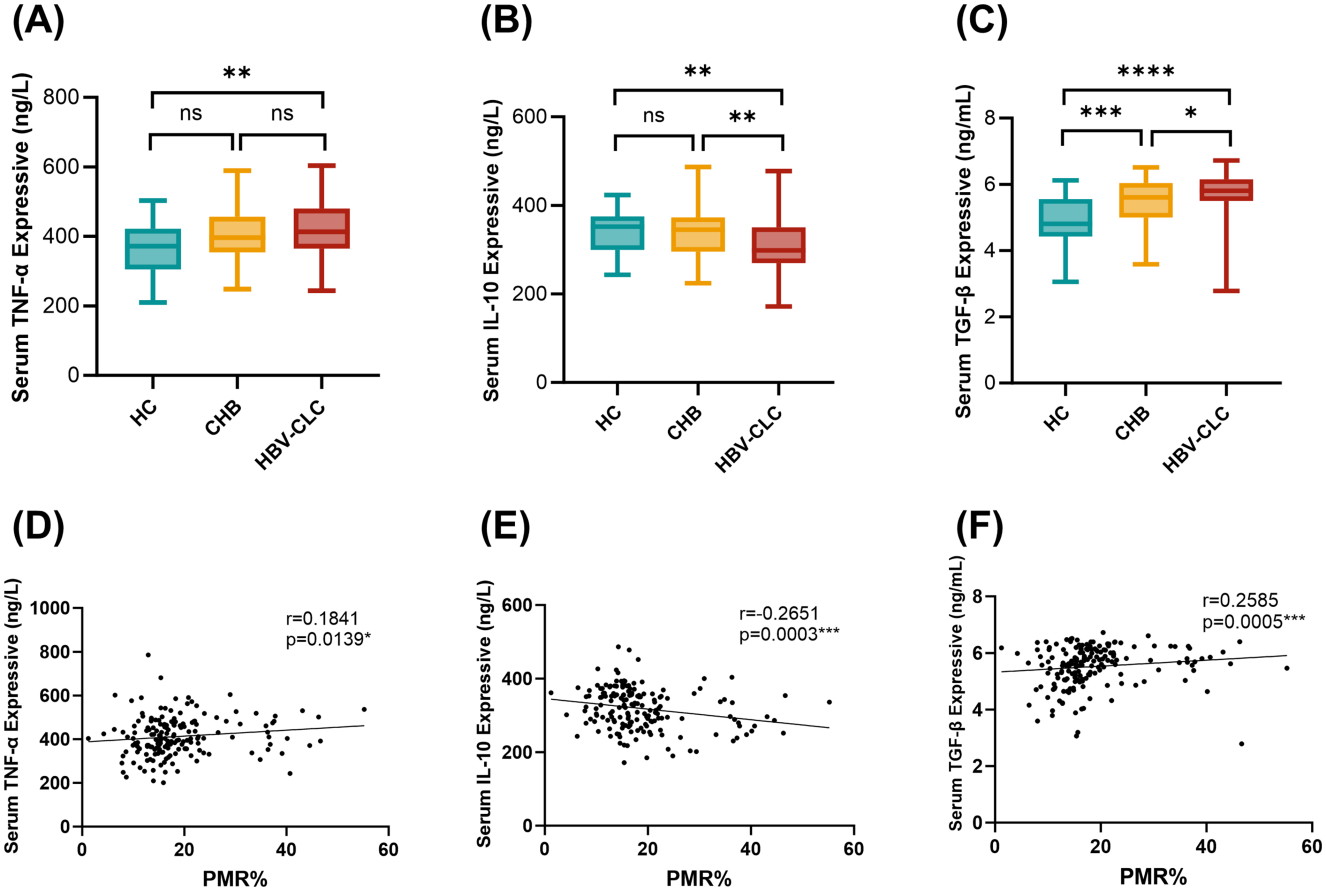
Serum TNF-α, IL-10, and TGF-β levels in LC, CHB, and HCs patients. **(A)** Elisa showing that serum TNF-α levels were significantly higher in patients with HBV-CLC (413.2 [365.1–480.3] ng/L) as compared to HC groups (371.8 [305.4–421.7] ng/L). However, there was no significant difference in serum TNF-α expression between HBV-CLC patients and CHB patients (396.7 [354.2–456.7] ng/L). **(B)** IL-10 levels were markedly reduced in HBV-CLC patients (298.3 [269.8–350.3] ng/L) relative to CHB (344.9 [295.6–372.9] ng/L) and HC (352.4 [299.8–375.1] ng/L). **(C)** Serum TGF-β was significantly overexpressed in HBV-CLC (5.811 [5.499–6.155] ng/mL) compared with HC (4.807 [4.424–5.552] ng/mL) and CHB (5.606 [5.000–6.039] ng/mL). **(D–F)** Relationships between RNF41 methylation level with TNF-α, IL-10, and TGF-β expressive in serum were analyzed with Spearman correlation analysis. Statistical analysis of TNF-α, IL-10, and TGF-β levels **(A–C)** based on the Median [lower quartiles-upper quartiles]. p values were obtained using Student’s t test. *p < 0.05, **p ≤ 0.01, ***p ≤ 0.001, ****p ≤ 0.0001.

Peripheral blood serum levels of inflammatory cytokines TNF-α, IL-10, and TGF-β were quantitatively assessed using ELISA, with results presented in Figure 4. HBV-CLC patients exhibited significantly elevated TNF-α concentrations compared to HC groups. However, there was no significant difference in serum TNF-α expression between HBV-CLC patients and CHB patients, although there was an increasing trend in the HBV-CLC group (Figure 4A). Conversely, IL-10 levels were markedly reduced in HBV-CLC patients relative to CHB and HC (Figure 4B). We also examined the expression of TGF-β, a pro-fibrotic cytokine. Serum TGF-β was significantly overexpressed in HBV-CLC compared with HC and CHB (Figure 4C). Spearman correlation analysis revealed associations between RNF41 promoter methylation level in PBMCs and cytokine profiles (Figures 4D–F): weak positive correlation with TNF-α (*r* = 0.1841, *p* = 0.0139) and TGF-β (*r* = 0.2585, *p* = 0.0005), and weak negative correlation with IL-10 (*r* = −0.2651, *p* = 0.0003), suggesting epigenetic regulation of inflammatory responses during cirrhosis progression.

### 3.5 Diagnostic value of RNF41 promoter methylation level

**Figure 5:**
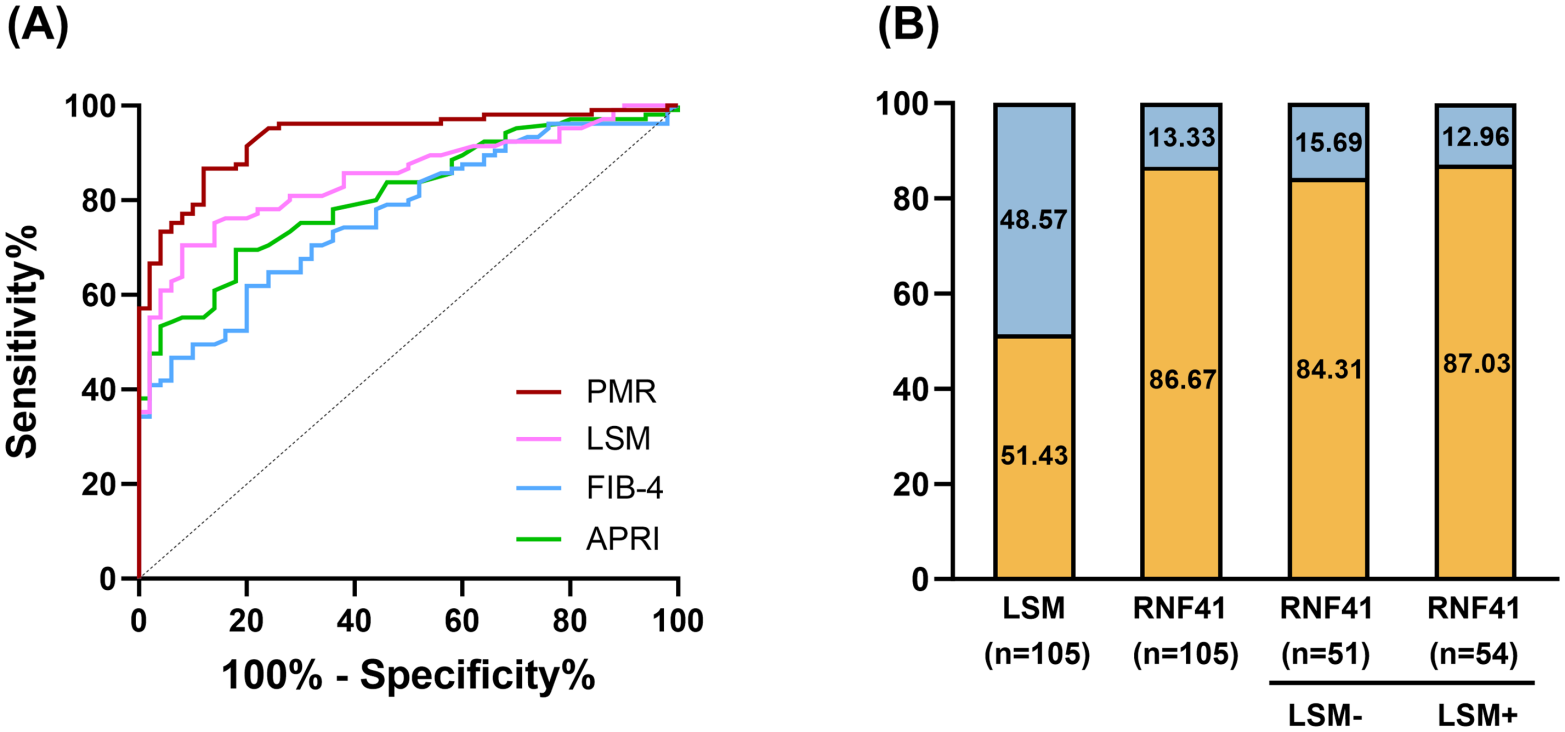
Diagnostic value of RNF41 promoter methylation level in PBMC in HBV-associated compensatory cirrhosis. **(A)** ROC curves of RNF41 methylation level, LSM level, FIB-4 Score, and APRI Score in discriminating HBV-associated compensatory LC from CHB. PMR values of the RNF41 promoter showed an AUC of 0.932, higher than that of LSM (AUC = 0.848), FIB-4 Score (AUC = 0.843), and APRI (AUC = 0.845). The optimal cut-off PMR of 16.138% was selected. **(B)** Rate of positive results for LSM, PBMCs’ RNF41 promoter methylation levels in patients with HBV-associated liver cirrhosis, and for RNF41 by LSM status.

Receiver operating characteristic (ROC) curve analysis (Figure 5A) demonstrated superior diagnostic performance of PMR value of RNF41 promoter compared to conventional non-invasive indices, including LSM, FIB-4 Score and APRI Score. At the optimal cutoff value of 16.138%, RNF41 methylation achieved a sensitivity of 86.67% and specificity of 81.00% (Table 3), indicating robust diagnostic accuracy for cirrhosis detection. Among 105 HBV-CLC patients, RNF41 promoter hypermethylation was more prevalent than elevated LSM (91 vs. 54 cases). Notably, 43 of 51 patients (84.31%) without elevated LSM showed RNF41 promoter hypermethylation, comparable to the hypermethylation rate in patients with elevated LSM (47 of 54 cases, 87.03%) (Figure 5B). These findings suggest that RNF41 methylation status may provide additional diagnostic value beyond LSM measurements.

**Table 3:** Diagnostic values of RNF41 methylation level, LSM, FIB-4 Score, and APRI Score for distinguishing HBV-associated compensatory LC from CHB.

Parameter	Sensitivity	Specificity	Youden index	AUC	95%CI
PMR	86.67%	88.00%	0.747	0.932	0.893–0.972
LSM	70.48%	92.00%	0.625	0.848	0.789–0.908
FIB-4	61.90%	80.00%	0.419	0.767	0.693–0.841
APRI	69.52%	92.00%	0.515	0.809	0.742–0.875

### 3.6 Independent risk factors for HBV-associated compensated liver cirrhosis

Univariate and multivariate analyses were performed to identify independent risk factors for HBV-CLC. As presented in Table 4, eight variables with *p* < 0.05 in univariate regression (RNF41 PMR, ALT, AST, TBIL, ALB, PTA, PLT, LSM) were included in multivariate analysis. Multivariate regression revealed RNF41 PMR (OR = 1.670, 95%CI:1.302–2.142, *p* < 0.001) and LSM (OR = 1.455, 95%CI:1.120–1.889, *p* = 0.005) as independent risk factors for HBV-CLC.

**Table 4:** Independent risk factors for the development of HBV-associated compensated liver cirrhosis.

Variables	Univariate analysis		Multivariate analysis	
	OR (95% CI)	*p* Value	OR (95% CI)	*p* Value
RNF41 PMR	1.689 (1.386–2.059)	<0.001^****^	1.670 (1.302–2.142)	<0.001^****^
Age	1.028 (0.992–1.065)	0.130	–	–
Gender (Male)	1.227 (0.604–2.495)	0.572	–	–
Log10[HBV DNA]	0.798 (0.507–1.255)	0.329	–	–
HBsAg	1.000 (1.000–1.000)	0.202	–	–
HBeAg (+)	0.848 (0.429–1.677)	0.636	–	–
ALT	1.032 (1.003–1.062)	0.028^*^	0.984 (0.915–1.058)	0.667
AST	1.106 (1.049–1.167)	<0.001^****^	1.080 (0.920–1.269)	0.345
TBIL	1.117 (1.050–1.188)	<0.001^****^	1.026 (0.913–1.154)	0.658
ALB	0.892 (0.806–0.988)	0.028^*^	1.061 (0.823–1.368)	0.646
PTA	0.936 (0.906–0.967)	<0.001^****^	0.976 (0.914–1.043)	0.482
PLT	0.987 (0.982–0.994)	<0.001^****^	0.991 (0.979–1.002)	0.118
AFP	1.179 (0.994–1.398)	0.059	–	–
LSM	1.474 (1.277–1.704)	<0.001^****^	1.455 (1.120–1.889)	0.005^***^

*p < 0.05, ***p < 0.005, ****p < 0.001.

## 4 Discussion

Our study demonstrates that RNF41 promoter methylation levels in PBMCs are significantly elevated in HBV-CLC patients compared to both HCs and CHB patients. As a non-invasive hematologic biomarker for early cirrhosis, RNF41 methylation has shown better diagnostic performance than traditional indicators such as LSM, FIB-4, and APRI scores. Notably, RNF41 maintained high hypermethylation rates (60%) in HBV-CLC patients without elevated LSM, indicating its complementary diagnostic value to LSM.

Previous studies have identified downregulation of RNF41 in macrophages from both human cirrhotic livers and murine fibrotic models (18). While these investigations established RNF41’s role in hepatic fibrosis through mechanisms involving macrophage phenotype switching via the C/EBP-β/PPAR-γ pathway and subsequent IGF-1-mediated HSC inactivation, they were limited to liver tissue analyses. Importantly, no prior study has examined RNF41 alterations in PBMCs—a clinically accessible cell population. Our work addresses this critical gap by demonstrating significant RNF41 promoter hypermethylation in PBMCs from HBV-CLC patients, while systematically evaluating its diagnostic potential as a non-invasive biomarker. This circulating epigenetic offers new possibilities for non-invasive peripheral blood-based fibrosis monitoring.

Macrophages are the most abundant non-parenchymal cells (NPCs) in the liver, playing pivotal roles throughout the progression of liver diseases—from initial tissue injury to chronic inflammation, fibrosis, and eventual repair (27, 28). Macrophages exhibit phenotypic plasticity with two polarization states (29): classically activated pro-inflammatory M1, characterized by high expression of cytokines (TNF-α, IL-6, IL-1β), and alternatively activated immunoregulatory M2, associated with anti-inflammatory mediators (IL-10, TGF-β) promoting tissue repair. Although it remains challenging to categorically classify these macrophage subsets as strictly pro-fibrotic or anti-fibrotic, accumulating evidence demonstrates that distinct macrophage subpopulations coexist in the liver and play stage-specific roles throughout the progression of hepatic fibrosis (28). Bao-Ming Wu et al. found that Margotoxin alleviates liver fibrosis by promoting M2 macrophage polarization through the STAT1/STAT6 signaling pathway, reducing pro-inflammatory cytokine secretion while increasing the production of the anti-inflammatory cytokine IL-10 (30). Previous studies by Moreno-Lanceta et al. (18) demonstrated that in liver macrophages, RNF41 upregulation stimulates IL-10 production (positive correlation) while suppressing TGF-β expression (negative correlation). However, the correlation with TNF-α is not clear. In our study, PBMC analysis revealed parallel but distinct patterns: TNF-α and TGF-β expression was significantly elevated in HBV-CLC patients and showed a positive correlation with RNF41 methylation levels in PBMC, while IL-10 expression was reduced and negatively correlated with RNF41 methylation. These findings are consistent with the previous research conducted in the liver and suggest that RNF41 may be involved in the development of HBV-associated cirrhosis inflammation and fibrosis by regulating macrophage polarization. However, while we assessed cytokine levels, the precise molecular mechanisms underlying the interplay between RNF41 and these cytokines remain to be elucidated.

Compared to traditional methods measuring plasma protein levels, DNA methylation occurs upstream of protein translation, offering earlier detection capabilities and more timely reflection of gene expression regulation. In this study, we demonstrated for the first time that RNF41 methylation levels in PBMCs are significantly elevated in HBV-CLC patients compared to both CHB and HC groups, and systematically evaluated the diagnostic potential of RNF41 methylation as a non-invasive biomarker for HBV-related early liver cirrhosis. However, several limitations should be acknowledged: (1) the relatively small sample size and single-center design may introduce selection bias; (2) while we assessed inflammatory cytokine profiles, the precise molecular mechanisms linking RNF41 to hepatic fibrogenesis remain to be fully elucidated. Meanwhile, aberrant methylation of the RNF41 promoter may occur in specific cell components within PBMCs. In future work, we plan to analyze methylation changes in specific PBMC subpopulations.

In summary, RNF41 promoter hypermethylation in PBMCs represents a characteristic epigenetic alteration in HBV-associated liver cirrhosis patients. The diagnostic performance of RNF41 PMR values in distinguishing HBV-CLC from CHB significantly outperformed conventional indices including LSM, FIB-4, and APRI scores, suggesting that RNF41 methylation status in PBMCs may serve as a promising non-invasive diagnostic biomarker for early cirrhosis. However, the precise role of RNF41 in LC pathogenesis and its potential as a therapeutic target and prognostic indicator warrant further investigation.

## Data Availability

The raw data supporting the conclusions of this article will be made available by the authors, without undue reservation.

## Glossary

LC: liver cirrhosis
RNF41: Ring finger protein 41
Nrdp1: neuregulin receptor degradation protein 1
HBV: hepatitis B virus
HBV-CLC: HBV-related compensated liver cirrhosis
CHB: chronic hepatitis B
HC: healthy controls
PBMC: peripheral blood mononuclear cell
ROC: receiver operating characteristic
AUC: area under the curve
TE: transient elastography
LSM: liver stiffness measurement
APRI: aspartate aminotransferase-to-platelet ratio index
FIB-4: fibrosis-4
HSC: hepatic stellate cell
MMP-9: matrix metalloproteinase-9
IGF-1: insulin-like growth factor-1
TNF-α: tumor necrosis factor-α
IL-6: interleukin-6
HBsAg: Hepatitis B surface antigen
INR: international normalized ratio
PMR: methylated reference value
ALT: alanine aminotransferase
AST: aspartate aminotransferase
TBIL: total bilirubin
ALB: albumin
PTA: prothrombin time activity
AFP: alpha-fetoprotein
IL-10: Interleukin-10
TGF-β: transforming growth factor-β

## References

[1] Ginès P Krag A Abraldes JG Solà E Fabrellas N Kamath PS. Liver cirrhosis. Lancet (London, England). (2021) 398:1359–76. doi: 10.1016/S0140-6736(21)01374-X 34543610

[2] Caligiuri A Gentilini A Pastore M Gitto S Marra F. Cellular and molecular mechanisms underlying liver fibrosis regression. Cells. (2021) 10:2759. doi: 10.3390/cells10102759, PMID: 34685739 PMC8534788

[3] Huang DQ Terrault NA Tacke F Gluud LL Arrese M Bugianesi E. Global epidemiology of cirrhosis - aetiology, trends and predictions. Nat Rev Gastroenterol Hepatol. (2023) 20:388–98. doi: 10.1038/s41575-023-00759-2, PMID: 36977794 PMC10043867

[4] Marcellin P Gane E Buti M Afdhal N Sievert W Jacobson IM. Regression of cirrhosis during treatment with tenofovir disoproxil fumarate for chronic hepatitis B: a 5-year open-label follow-up study. Lancet (London, England). (2013) 381:468–75. doi: 10.1016/S0140-6736(12)61425-1 23234725

[5] Schuppan D Surabattula R Wang XY. Determinants of fibrosis progression and regression in NASH. J Hepatol. (2018) 68:238–50. doi: 10.1016/j.jhep.2017.11.012, PMID: 29154966

[6] Gidener T Ahmed OT Larson JJ Mara KC Therneau TM Venkatesh SK. Liver stiffness by magnetic resonance elastography predicts future cirrhosis, decompensation, and death in NAFLD. Clin Gastroenterol Hepatol. (2021) 19:1915–1924.e6. doi: 10.1016/j.cgh.2020.09.044, PMID: 33010409 PMC9096913

[7] Niu L Thiele M Geyer PE Rasmussen DN Webel HE Santos A. Noninvasive proteomic biomarkers for alcohol-related liver disease. Nat Med. (2022) 28:1277–87. doi: 10.1038/s41591-022-01850-y, PMID: 35654907 PMC9205783

[8] Takyar V Etzion O Heller T Kleiner DE Rotman Y Ghany MG. Complications of percutaneous liver biopsy with Klatskin needles: a 36-year single-Centre experience. Aliment Pharmacol Ther. (2017) 45:744–53. doi: 10.1111/apt.13939, PMID: 28074540 PMC5290209

[9] Ozturk A Olson MC Samir AE Venkatesh SK. Liver fibrosis assessment: MR and US elastography. Abdominal Radiol (New York). (2022) 47:3037–50. doi: 10.1007/s00261-021-03269-4, PMID: 34687329 PMC9033887

[10] Bai X Pu C Zhen W Huang Y Zhang Q Li Z. Identifying liver cirrhosis in patients with chronic hepatitis B: an interpretable machine learning algorithm based on LSM. Ann Med. (2025) 57:2477294. doi: 10.1080/07853890.2025.2477294 40104981 PMC11924261

[11] Kjaergaard M Lindvig KP Thorhauge KH Andersen P Hansen JK Kastrup N. Using the ELF test, FIB-4 and NAFLD fibrosis score to screen the population for liver disease. J Hepatol. (2023) 79:277–86. doi: 10.1016/j.jhep.2023.04.002, PMID: 37088311

[12] Graupera I Thiele M Serra-Burriel M Caballeria L Roulot D Wong GL-H. Low accuracy of FIB-4 and NAFLD fibrosis scores for screening for liver fibrosis in the population. Clin Gastroenterol Hepatol. (2022) 20:2567–2576.e6. doi: 10.1016/j.cgh.2021.12.034, PMID: 34971806

[13] Itakura J Kurosaki M Setoyama H Simakami T Oza N Korenaga M. Applicability of APRI and FIB-4 as a transition indicator of liver fibrosis in patients with chronic viral hepatitis. J Gastroenterol. (2021) 56:470–8. doi: 10.1007/s00535-021-01782-3 33791882

[14] Chen N Sun Y Luo P Tang Y Fan Y Han L. Association of CXCR4 gene expression and promoter methylation with chronic hepatitis B-related fibrosis/cirrhosis. Int Immunopharmacol. (2024) 139:112686. doi: 10.1016/j.intimp.2024.112686, PMID: 39053226

[15] Li K Qin L Jiang S Li A Zhang C Liu G. The signature of HBV-related liver disease in peripheral blood mononuclear cell DNA methylation. Clin Epigenetics. (2020) 12:81. doi: 10.1186/s13148-020-00847-z, PMID: 32513305 PMC7278209

[16] Wauman J De Ceuninck L Vanderroost N Lievens S Tavernier J. RNF41 (Nrdp1) controls type 1 cytokine receptor degradation and ectodomain shedding. J Cell Sci. (2011) 124:921–32. doi: 10.1242/jcs.078055, PMID: 21378310 PMC3115735

[17] Pearson G Soleimanpour SA. A ubiquitin-dependent mitophagy complex maintains mitochondrial function and insulin secretion in beta cells. Autophagy. (2018) 14:1160–1. doi: 10.1080/15548627.2018.1446627, PMID: 29799764 PMC6103716

[18] Moreno-Lanceta A Medrano-Bosch M Fundora Y Perramón M Aspas J Parra-Robert M. RNF41 orchestrates macrophage-driven fibrosis resolution and hepatic regeneration. Sci Transl Med. (2023) 15:eabq6225. doi: 10.1126/scitranslmed.abq6225, PMID: 37437019 PMC10712730

[19] Wang C Chen T Zhang J Yang M Li N Xu X. The E3 ubiquitin ligase Nrdp1 'preferentially' promotes TLR-mediated production of type I interferon. Nat Immunol. (2009) 10:744–52. doi: 10.1038/ni.1742, PMID: 19483718

[20] Meng Z Xu R Xie L Wu Y He Q Gao P. A20/Nrdp1 interaction alters the inflammatory signaling profile by mediating K48- and K63-linked polyubiquitination of effectors MyD88 and TBK1. J Biol Chem. (2021) 297:100811. doi: 10.1016/j.jbc.2021.100811 34023381 PMC8233150

[21] Zhang J Liu Y Chen H Yuan Q Wang J Niu M. MyD88 in hepatic stellate cells enhances liver fibrosis via promoting macrophage M1 polarization. Cell Death Dis. (2022) 13:411. doi: 10.1038/s41419-022-04802-z, PMID: 35484116 PMC9051099

[22] Meng H Cao Y Qin J Song X Zhang Q Shi Y. DNA methylation, its mediators and genome integrity. Int J Biol Sci. (2015) 11:604–17. doi: 10.7150/ijbs.11218, PMID: 25892967 PMC4400391

[23] Nishiyama A Nakanishi M. Navigating the DNA methylation landscape of cancer. Trends Genet TIG. (2021) 37:1012–27. doi: 10.1016/j.tig.2021.05.002, PMID: 34120771

[24] Horvath S Raj K. DNA methylation-based biomarkers and the epigenetic clock theory of ageing. Nat Rev Genet. (2018) 19:371–84. doi: 10.1038/s41576-018-0004-3, PMID: 29643443

[25] Jones PA. Functions of DNA methylation: islands, start sites, gene bodies and beyond. Nat Rev Genet. (2012) 13:484–92. doi: 10.1038/nrg3230 22641018

[26] Yang L Liu Y Sun Y Huang C Li J Wang Y. New advances of DNA/RNA methylation modification in liver fibrosis. Cell Signal. (2022) 92:110224. doi: 10.1016/j.cellsig.2021.110224, PMID: 34954394

[27] Casari M Siegl D Deppermann C Schuppan D. Macrophages and platelets in liver fibrosis and hepatocellular carcinoma. Front Immunol. (2023) 14:1277808. doi: 10.3389/fimmu.2023.1277808, PMID: 38116017 PMC10728659

[28] Wang C Ma C Gong L Guo Y Fu K Zhang Y. Macrophage polarization and its role in liver disease. Front Immunol. (2021) 12:803037. doi: 10.3389/fimmu.2021.803037, PMID: 34970275 PMC8712501

[29] Tacke F Zimmermann HW. Macrophage heterogeneity in liver injury and fibrosis. J Hepatol. (2014) 60:1090–6. doi: 10.1016/j.jhep.2013.12.025, PMID: 24412603

[30] Wu B-M Liu J-D Li Y-H Li J. Margatoxin mitigates CCl4-induced hepatic fibrosis in mice via macrophage polarization, cytokine secretion and STAT signaling. Int J Mol Med. (2020) 45:103–14. doi: 10.3892/ijmm.2019.4395, PMID: 31746414 PMC6889929

